# A non-invasive tool for the early identification of children at risk of cardiometabolic dysfunction: data from the PODiaCar project

**DOI:** 10.1038/s41390-025-04603-y

**Published:** 2025-12-03

**Authors:** Valeria Calcaterra, Lucia Labati, Cristina Campoy, Virginia Rossi, Giulia Fiore, Mireia Escudero-Marin, Matteo Vandoni, Elvira Verduci, Luca Marin, Valter Pagani, Camilo Corbellini, Savina Mannarino, Rocio Bonillo Leon, Inmaculada Guerrero, Vittoria Carnevale Pellino, Alessandro Gatti, Umberto Ciriello, Gianvincenzo Zuccotti

**Affiliations:** 1 Department of Internal Medicine and Therapeutics, University of Pavia, Pavia, Italy; 2 Pediatric Department, Buzzi Children’s Hospital, Milano, Italy; 3 Department of Biomedical and Clinical Science, University of Milano, Milano, Italy; 4 Department of Paediatrics, University of Granada, Granada, Spain; 5 Instituto Biosanitario de Granada - IBS-18016, Granada, Spain; 6 Laboratory of Adapted Motor Activity (LAMA), Department of Public Health, Experimental Medicine and Forensic Science, University of Pavia, Pavia, Italy; 7 Department of Health Sciences, University of Milano, Milano, Italy; 8 Grant & Research Department-LJA-2021, Asomi College of Sciences, Marsa, Malta; 9 Department of Physiotherapy, LUNEX International University of Health, Exercise and Sports, Differdange, Luxembourg; 10 Luxembourg Health & Sport Sciences Research Institute A.S.B.L., Differdange, Luxembourg; 11 Pediatric Cardiology Buzzi Children’s Hospital, Milano, Italy; 12 National PhD Programme in One Health approaches to infectious diseases and life science research, Department of Public Health, Experimental and Forensic Medicine, University of Pavia, Pavia, Italy

## Abstract

**Background:**

Early identification of children at risk for metabolic syndrome (MetS) can reveal traits linked to cardiometabolic disease. We aimed to develop a simple, user-friendly tool to detect pediatric cardiometabolic risk using clinical, nutritional, and lifestyle data.

**Methods:**

A total of 317 patients (11.35 ± 3.62) were assessed using clinical, dietary, and biochemical data. Metabolic risk was defined by a MetS z-score >0.75, and MetS diagnosis required at least three altered parameters (body composition, blood pressure, glucose, lipids). A 22-variable binary tool generated a cumulative risk score: ≥7 altered components indicated high risk; otherwise, low risk.

**Results:**

A pathological MetS-score was found in 62.15% of subjects, while MetS was diagnosed in 39.4%. The MetS z-score was significantly correlated with MetS prevalence (*r* = 0.581). When considering a screening tool score ≥7, along with patients presenting at least 3 of 4 altered MetS parameters, the results demonstrated good sensitivity (0.768 [0.715, 0.835]), negative predictive value (0.775 [0.702, 0.848]), and accuracy (0.618 [0.564, 0.672]), though specificity (52.1% [0.420, 0.600]) and positive predictive value (0.511 [0.439, 0.582]) were moderate.

**Conclusion:**

A score ≥7 reliably identifies children at cardiometabolic risk, providing a sensitive, non-invasive tool that supports early detection, prevention, and personalized care while reducing time and healthcare costs.

**Impact:**

- Early detection of at-risk children can uncover cardio-metabolic traits.
- A 22-noninvasive variable tool was developed to identify pediatric cardio-metabolic risk.
- A score ≥7 effectively identifies children at cardiometabolic risk.
- The proposed non-invasive tool achieves good sensitivity (76.8%) and moderate specificity (52.1%).
- The tool supports clinicians in prevention, monitoring, and personalized care.

## Introduction

Non-communicable diseases pose a significant public health challenge in pediatrics, both now and in the future.^1^ The pediatric population represents the cornerstone of life-course approaches for the prevention, management, and treatment of NCDs.^1,2^

According to the World Health Organization (WHO), the risk of cardiovascular disease (CVD) and type 2 diabetes (T2D) arises from a combination of risk factors that begin in the prenatal period and continue through childhood and adulthood^3^ Specifically, the National Institutes of Health (NIH) classifies CVD risk factors into two main categories: non-modifiable (such as age, sex, race/ethnicity, familial and genetic predispositions, congenital conditions, and socioeconomic status) and modifiable factors. The latter can be further divided into cardiometabolic factors (e.g., hypertension, diabetes, dyslipidemia) and lifestyle factors (e.g., physical inactivity, poor diet, and obesity).^4,5^

While CVD and T2D are uncommon during childhood, early risk factors are often identifiable from a young age. The progression toward these conditions occurs along a continuum, becoming more pronounced as risk factors accumulate.^6^ These factors often interact and amplify one another, progressively leading to metabolic derangement, endothelial damage, vascular and myocardial remodeling, and the onset of atherosclerotic processes.^4^ Therefore, early detection of these interconnected variables, along with timely intervention on modifiable risk factors, is crucial to protecting pediatric health.

Early identification of children at risk for metabolic syndrome (MetS) can reveal individual traits linked to cardio-metabolic disease development and treatment outcomes.^7,8^ MetS refers to a combination of cardio-metabolic risk factors, including visceral obesity, high blood pressure, abnormal lipid levels, and impaired glucose regulation, that collectively heighten the risk of CVD and T2D.^9^ Its prevalence in youth varies widely, from 0.2% to 38.9%, and is estimated at 3.3% in the general pediatric population. Rates increase significantly in those with excess weight: 11.9% in children with overweight and 29.2% in those with obesity.^10^ The condition often reflects obesity trends, especially in high-income countries, and its distribution also depends on variables like age, sex, ethnicity, and diagnostic standards used.

Although there is consensus on the distinctive features of MetS, no international diagnostic criteria currently exist for the pediatric population.^11^ Recently, Gurka et al.^12^ proposed a metabolic score (MetS z-score) as a highly sensitive and specific tool for detecting risk markers of MetS, which was also found to be strongly associated with the incidence of T2D.^13^ The MetS z-score is a dynamic index useful for monitoring changes over time within a population and for identifying differences in the rate of change based on clinical status.

Several tools have been proposed to screen metabolic disorders in children, ranging from simple anthropometric indices (e.g. waist circumference or waist-to-height ratio) to more complex composite index (e.g. visceral adiposity index or body shape index, triponderal mass index, conicity index).^14–17^ Although simple anthropometric indices can provide useful information, their accuracy is limited because they mainly capture current body size and adiposity, without considering the metabolic and environmental factors that shape long-term cardiometabolic risk. Early-life influences, including parental health status, intrauterine environment, and fetal growth patterns, play a pivotal role in these processes^6,11,18,19^ and may not be reflected in conventional anthropometric measures. Accounting for early-life and contextual factors enhances risk estimation by capturing latent susceptibilities and developmental influences beyond cross-sectional body measurements. An integrated approach thus allows for more accurate and individualized cardiometabolic risk stratification, particularly during growth and maturation.

The aim of this study was to develop a simple, practical, and user-friendly tool to detect at cardio-metabolic risk (CMR) pediatric patients based on clinical features, medical history, nutrition, and lifestyle behaviors. We used the MetS z-score to stratify at-risk patients and the prevalence of MetS to assess the accuracy of the tool in detecting it. A high-quality risk score for cardiometabolic disorders in toddlers and young children could significantly improve awareness and enhance the guidance pediatricians offer to both parents and children to prevent CVD and to personalize care and promote every child’s right to health.

## Methods

### Study design

The study is a cross-sectional analysis, part of the “Fight against Pediatric Obesity: from a predictive tool for type 2 Diabetes and Cardiovascular diseases risk to healthy educational programs (PODiaCar)” european project (101128946-PODiaCar-EU4H-2022-PJ-3). PODiaCar (www.podiacar.eu) is a project aimed at addressing childhood obesity and its related complications, such as type 2 diabetes and cardiovascular diseases. It through multidisciplinary collaboration and digital innovation promotes a proactive, data-driven approach to child health and disease prevention. The project aligns with the European Commission’s “Healthier Together” initiative, targeting significant non- communicable diseases (NCDs) and is an initiative within the strategic framework of the European Digital and Health Executive Agency (HaDEA). The project team is composed of Buzzi’s Children Hospital, Milano, Italy (coordinator); University of Pavia, Pavia (Italy); University of Granada, Granada, Spain; Asomi College of Sciences, Marsa, Malta; LUNEX International University of Health, Exercise and Sports, Differdange, Luxembourg.

The study protocol received approval from the Ethics Committee of Lombardia 1 (protocol number CET 202–2023). It was conducted in accordance with the Helsinki Declaration of 1975, as revised in 2008. Before participation, both children and their parents were thoroughly informed about the study’s aims and procedures. Verbal and/or written assent was obtained from the children according to age, while written informed consent was signed by their parents or legal guardians.

### Participants

We consecutively included 317 patients (aged between 3 and 18 years) of both sexes who were referred to the Vittore Buzzi Children’s Hospital (Milano, Italy) and University of Granada (Granada, Spain) by their general practitioner or primary care pediatrician for auxological assessment related to weight management, between November 2023 and March 2025.

Exclusion criteria were any genetic syndromes known to be usually associated with obesity (e.g., Prader–Willi, Bardet–Biedl), any ongoing medical therapy, and concomitant chronic or acute illnesses.

Historical data, clinical evaluation, dietary patterns, and biochemical profiles were considered for all participants to create the screening tool and define the MetS severity z-score and MetS prevalence. To minimize potential bias during evaluations and data collection, study staff provided comprehensive guidance to all individuals responsible for assessing outcomes.

### Historical data

Evaluation of the anamnestic data include:

- Family history, assessed through interview with the parents, was considered positive when the presence of obesity in parents and diabetes mellitus and hypertension in parents and grandparents were reported.
- Socioeconomic status, determined according to parents’ level of education, occupation, and income.
- Maternal age, cardio-metabolic conditions (diabetes, obesity, hypertension) and maternal weight gain during pregnancy.
- Type of delivery (vaginal or cesarean section)
- Maternal and/or paternal exposure to environmental factors during pregnancy (smoke, alcohol consumption, stress, environmental disruptors)
- Neonatal data. On the basis of gestational age and birth weight, the children were defined appropriate for gestational age with a birth weight ≥10th percentile, small for gestational age with a birth weight <10th percentile, and large for gestational age with a birth weight >90th.^20^
- Early life nutritional data (breast/formula/cow’s milk; timing of complementary feeding).
- Infants’ antibiotics exposure.
- Inappropriate bottle use, defined as children over 12 months of age drinking ≥2 bottle-type containers/day.
- Sleep duration, assessed according to age-specific WHO guidelines.^21^
- Physical activity level and/or sedentariness, assessed according to age-specific WHO guidelines.^21^

### Clinical evaluation

In all participants, height (Ht), weight, pubertal stage, waist circumference (WC), WC/Ht, body mass index (BMI) were recorded. Systolic (SBP) and diastolic (DBP) blood pressure measurements were also collected.

Standing Ht was assessed using a Harpenden stadiometer equipped with a fixed vertical backboard and an adjustable headpiece. The measurement was taken with the subject standing upright without shoes, heels together, toes apart, hands relaxed at their sides, and the head aligned in the Frankfort horizontal plane. Weight was recorded with participants wearing light clothing and no shoes, standing in the center of the scale platform while facing the recorder, with hands at their sides and eyes directed forward. Two measurements were obtained for each parameter, and a third measurement was taken if the difference between the initial readings exceeded 0.5 cm for Ht or 500 g for weight.

WC was measured in accordance with WHO guidelines, in the horizontal plane midway between the lowest ribs and the iliac crest.

Pubertal stage was clinically assessed according to Tanner’s criteria. Participants were classified into three groups: prepubertal (stage 1 = Tanner 1), middle puberty (stage 2 = Tanner 2–3), and late puberty (stage 3 = Tanner 4–5). Clinical evaluation of pubertal status was performed by trained physicians through inspection and palpation of secondary sexual characteristics (breast development in girls, genital development in boys, and pubic hair in both sexes). Pubertal development was included in the score as it represents a critical stage marked by transient insulin resistance and the combined influence of increased adiposity, hormonal changes, and metabolic alterations, all contributing to heightened cardiometabolic risk. Pubertal progression is closely linked to the consolidation of MS,^22^ making this stage a key window to improve risk stratification and to guide preventive interventions in the pediatric population.

BMI was calculated as body weight (kilograms) divided by Ht (meters squared) and was transformed into BMI z-scores using WHO reference values.

Blood pressure (BP) was assessed using a mercury sphygmomanometer after the participant had been seated comfortably for five minutes. An appropriately sized cuff was placed on the slightly flexed right arm at heart level, and two consecutive measurements were taken. The second reading was used for analysis.

### Assessment of dietary and lifestyle patterns

To assess adherence to the Mediterranean dietary pattern, the KIDMED Mediterranean Diet Quality Index questionnaire was administered. This tool consisted of 16 self-administered questions evaluating individuals’ dietary habits in relation to the Mediterranean diet. The KIDMED index provided a score ranging from 0 to 12, reflecting the level of adherence to the diet. Based on the total score, individuals were classified into three categories: (a) ≥8: optimal adherence to the Mediterranean diet (good); (b) 4–7: moderate adherence, indicating the need for dietary improvement (average); (c) ≤3: poor adherence, indicating very low diet quality (poor).^23^

Information on physical activity and sleep duration was obtained during the clinical anamnesis focused on children’s lifestyle, as reported by parents and/or caregivers. Data were then categorized according to the WHO age-specific guidelines^21^ for sleep and physical activity.

### Biochemical evaluation

Plasma glucose, insulin, triglycerides (TG), total, LDL and HDL cholesterol levels were evaluated. As a surrogate of insulin resistance (IR), homeostasis model analysis—insulin resistance (HOMA-IR) index and Triglyceride–glucose (TyG) index were calculated as following.

-HOMA-IR^24^ = (insulin × glucose)/22.5;
-TyG-index^25^= ln[fasting triglycerides (mg/dl)×fasting plasma glucose (mg/dl)/2]).

Dyslipidemia was defined by TC > 200 mg/dl and/or LDL-C > 130 mg/dl and/or TG > 150 mg/dl.^26^

To test the risk of MetS, we computed the MetS z-score as proposed by Gurka et al.^12^. Children were considered having MetS if the MetS z-score was higher than 0.75.

The formula to assess the MetS z-score is presented below:

Boys: MetS z-score = −4.931 + 0.2804 * BMI z-score - 0.0257 * HDL-C + 0.0189 * Systolic blood pressure +0.6240 * log(triglycerides) + 0.0140 * fasting glucose

Girls: MetS z-score = −4.3757 + 0.4849 * BMI z-score - 0.0176 * HDL-C + 0.0257 * Systolic blood pressure + 0.3172 * log(triglycerides) + 0.0083 * fasting glucose

The MetS z-score proposed by Gurka et al.^12^ offers several operational and methodological advantages over other scores, such as the IDEFICS score by Ahrens.^27^ It is derived via confirmatory factor analysis, yielding data-driven weights for each component (WC, triglycerides, HDL-C, BP, fasting glucose) and sex- and ethnicity-specific equations, which improves construct validity and comparability across subgroups. Practically, it does not require insulin, making it easier to apply where insulin is not measured. In addition, the Gurka score has shown longitudinal associations with future cardiometabolic outcomes, enabling continuous tracking of MetS severity over time.^28,29^

As previously reported,^30,31^ MetS was defined as the presence of at least three of the following risk factors:

- BMI z score ≥2 and/or WC/Ht >0.5^15^;
- SBP ≥ 130 mmHg and/or DBP ≥ 85 mmHg^32^;
- glycemia ≥ 100 mg/dL and/or HOMA-IR^24^ ≥ 2.5 if prepubertal stage 1 o ≥ 4 if pubertal stage 2, 3 and/or TryG index>7.88^25^;
- total cholesterol >200 mg/dl and/or cholesterol-HDL < 40 mg/dL in females and <50 mg/dL in males and/or triglycerides ≥ 100 mg/dL ( < 10 years) or ≥130 mg/dL ( ≥ 10 years).^26^

We used a pre-specified pediatric MetS definition based on fixed, clinically interpretable thresholds, rather than the percentile-based algorithms used for certain parameters in other pediatric classifications,^33–36^ to improve feasibility and cross-cohort comparability. In addition, because our cohort includes participants aged 3–18 years, frameworks restricted to narrower age bands (e.g., the International Diabetes Federation definition,^33^ for which a formal diagnosis is limited to 10–16 years; Cook et al.^35^ an adaptation of the National Centers for Environmental Prediction definition applied to adolescents aged 12–19 years; Ferranti et al^36^ developed using National Health and Nutrition Examination Survey data from adolescents aged 12–19 years) or derived from selected samples (e.g., the definition by Viner et al.^37^ is based on only 103 patients with obesity) or adults^38–40^ are suboptimal. Beyond BMI, we preferred to use the WHtR because it effectively identifies cardiometabolic risk in children^15^ and, compared with waist circumference alone, better tracks changes in abdominal adiposity during adolescence.^41^ Regarding glucose metabolism, since insulin resistance typically precedes overt dysglycemia in pediatric populations, we included HOMA-IR and/or the TyG index alongside fasting glucose.^9,11^ We did not use OGTT-based classification owing to the time burden, the limited reproducibility of the 2-hour glucose value in pediatrics, and its poor applicability in routine or large-scale settings.^42^ The euglycemic–hyperinsulinemic clamp remains the reference method for assessing insulin resistance; however, its invasive nature, time requirements, and complexity limit its use in pediatric practice.

### CMR screening score tool

Data based on 22 variables were used to create a tool for CMR assessment. Each parameter represents a dichotomous variable: for each of them, criteria have been defined to assign a score of 1 for a pathological situation or 0 for a normal condition, see Table 1 (score calculator in Supplementary Material 1).

**Table 1:** Parameters used to create cardiometabolic risk assessment.

Parameters	CMR Score
1. Gestational age<37 weeks	1
2. Presence of maternal cardiometabolic diseases during pregnancy	1
3. Exposure to environmental factors during pregnancy in mother	1
4. Exposure to environmental factors during pregnancy in father	1
5. Birth weight small or large for gestational age	1
6. Cesarean section	1
7. Gestational weight gain ( ≥ 10 kg)	1
8. Familiar/genetic risks for diabetes or hypertension	1
9. WC/Ht ratio >0.5	1
10. Adiposity rebound <6 years	1
11. BMI (z-score) ≥2	1
12. Pubertal stage >1	1
13. Low socioeconomic familiar status	1
14. Low adherence to MD_Kidmed ( ≤ 3 points)	1
15. Not exclusive breastfeeding	1
16. Timing of cow’s milk introduction <12 months	1
17. Timing of complementary feeding <6 months	1
18. Infants’ antibiotics exposure	1
19. Inappropriate bottle use	1
20. Sleep duration below age-specific WHO recommendations^15^	1
21. Inappropriate physical level or sedentariness assessed according to age-specific WHO guidelines^15^	1
22. Pathological blood pressure	1

*BMI* body mass index, *CMR* cardio-metabolic risk, *MD* mediterranean diet, *WC* waist circumference, *Ht* height.

The cumulative risk factors were summed to obtain a total risk score. To dichotomize the population, we selected a cut-off of seven dysregulated components, as this threshold corresponded to the median number of dysregulated components observed in individuals exhibiting a pathological MetS z-score. The median was selected as it represents a robust measure of central tendency, minimizes the influence of outliers, and ensures a balanced distribution of participants across risk groups. Accordingly, participants with ≥7 dysregulated components were classified as high risk, while those with fewer than 7 components were categorized as low risk.

### Statistical analysis

The Shapiro-Wilk test was used to assess the normality of quantitative variables. Since the quantitative variables followed a normal distribution, results were reported as mean values with standard deviations (SD). Categorical variables were presented as frequencies and percentages, and group comparisons were conducted using the unpaired t-test. In order to evaluate the relationship between the presence of a certain number of pathological variables and the MetS, a contingency table was constructed. As two dichotomous variables were considered, the tool score above/below the median and the presence or absence of at least 3 altered parameters characteristic of the MetS. To evaluate the level of significance through the contingency tables between these variables, the χ2-square test was used. To evaluate the tool’s accuracy in detecting MetS, sensitivity, specificity, positive predictive value (PPV), negative predictive value (NPV), accuracy, and likelihood ratios (LR) were calculated. Data analysis included the 95% confidence interval (95% CI).

Sample size and power calculations were performed a priori. Assuming a prevalence of 3.3% in the general pediatric population and 11.9% among children with overweight,^10^ with 95% power and a two-sided alpha of 0.05, the minimum sample size required was 243 participants. Given that our study included 317 children, the achieved statistical power under the same assumptions was greater than 99%.

All statistical analyses were performed using Microsoft Excel, with additional specific extensions (e.g., the Solver add-in) and advanced data analysis features enabled through the Data Analysis ToolPak.

## Results

We enrolled 317 patients (11.35 ± 3.62 years, range 2.95–17.62 years). Among them, 177 (55.83%) were males, and 140 (44.17%) were females. In Table 2, values of clinical parameters and biochemical data are reported.

**Table 2:** Clinical and biochemical parameters of the enrolled subjects, stratified by sex. Significance values for comparisons between subjects with high (score ≥7) and low (score <7) cardio-metabolic risk (CMR) are also reported.

	Males (*n* = 177)	Females (*n* = 140)	Total	*p*-value High vs Low CMR
Waist circumference (cm)	87.62 ± 19.66	82.85 ± 20.72	85.52 ± 13.89	0.003
Weight (kg)	66.98 ± 59.15	61.9 ± 20.95	64.75 ± 47.31	0.346
Height (cm)	150.72 ± 14.56	148.11 ± 15.01	149.57 ± 14.80	<0.001
Waist circumference/height ratio	0.58 ± 0.12	0.54 ± 0.13	0.56 ± 0.11	0.004
BMI (Body mass index [kg/m^2^])	26.97 ± 5.10	27.33 ± 5.87	27.13 ± 6.54	0.568
Body mass index (z-score)	2.69 ± 1.07	2.40 ± 1.07	2.56 ± 1.07	0.018
Pubertal stage (Tanner)				
Tanner 1	68 (38.42%)	56 (52.34%)	124 (43.66%)	-
Tanner 2 (2–3)	80 (45.20%)	32 (29.91%)	112 (39.44%)	-
Tanner 3 (4–5)	29 (16.38%)	19 (17.76%)	48 (16.90%)	-
Gestational age	38.91 ± 14.55	39.09 ± 10.27	39.00 ± 1.90	0.430
Systolic Blood Pressure (mmHg)	113.55 ± 53.13	110.21 ± 49.42	112.03 ± 12.14	0.038
Diastolic Blood Pressure (mmHg)	69.78 ± 32.42	68.98 ± 30.16	69.41 ± 8.59	0.481
Glycemia (mg/dl)	89.51 ± 34.81	85.92 ± 32.46	87.91 ± 9.54	0.002
Insulina (mcU/ml)	18.15 ± 17.71	19.20 ± 15.28	18.62 ± 16.88	0.634
HOMA	4.02 ± 4.15	4.23 ± 3.42	4.11 ± 3.95	0.697
TyG-index	8.26 ± 3.92	8.15 ± 3.97	8.21 ± 1.74	0.148
HbA1C (%)	5.30 ± 2.68	5.29 ± 2.65	5.29 ± 0.40	0.912
Total cholesterol (mg/dl)	157.72 ± 75.23	157.83 ± 71.67	157.77 ± 29.27	<0.001
LDL cholesterol (mg/dl)	85.86 ± 48.86	82.67 ± 44.16	84.41 ± 32.62	0.460
HDL cholesterol (mg/dl)	53.22 ± 31.03	58.24 ± 32.43	55.54 ± 23.49	<0.001
Tryglicerides (mg/dl)	97.84 ± 61.94	94.90 ± 64.02	96.53 ± 53.55	0.680

*BMI* body mass index, *CMR* cardio-metabolic risk, *HDL* high-density lipoprotein, *HOMA* homeostasis model analysis—insulin resistance, *LDL* low-density lipoprotein, *TyG* Triglyceride–glucose.

A pathological level of MetS-score was noted in 197 (62.15%) subjects. These subjects showed a median of 7 pathological parameters included in CMR score.

**Fig. 1:**
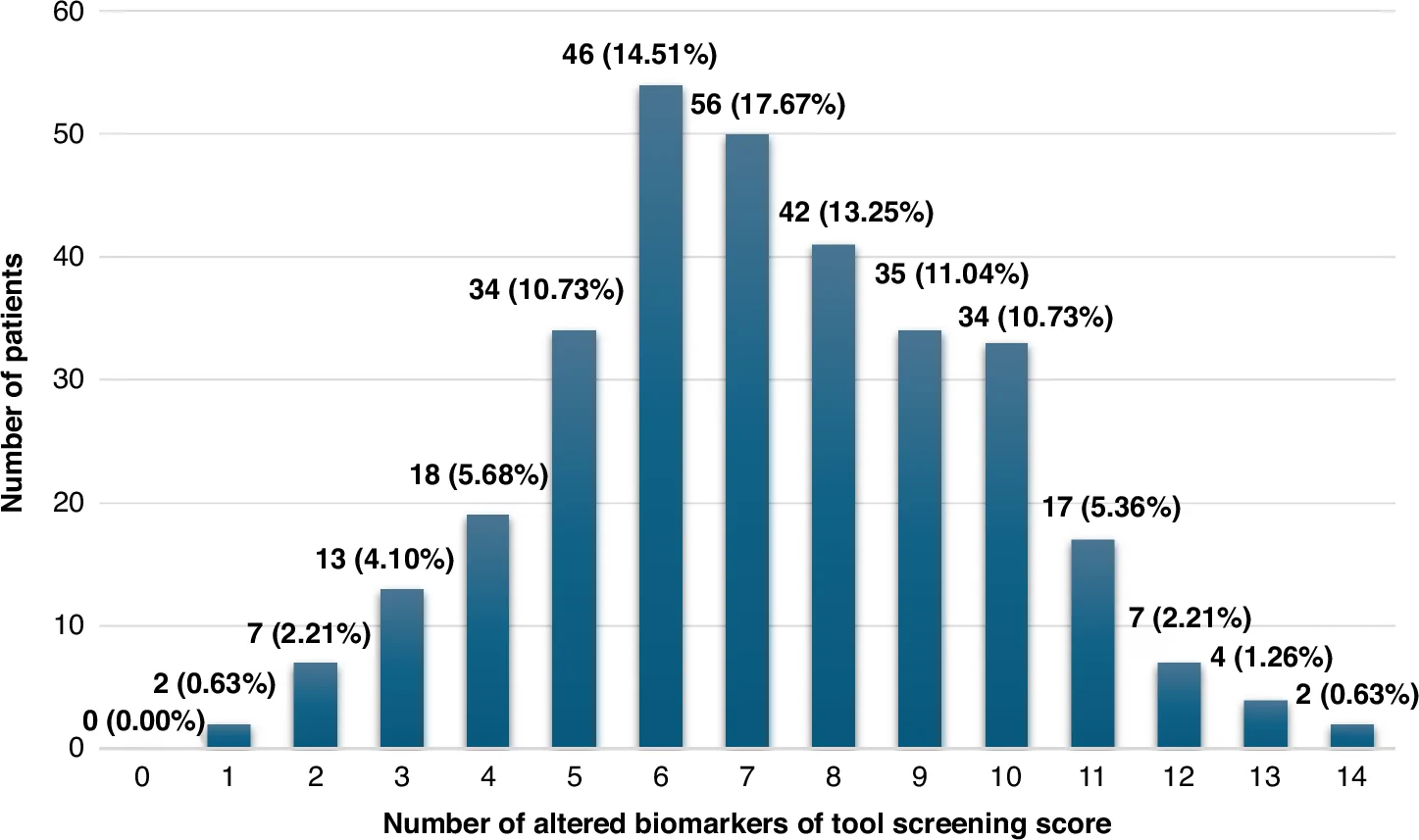
Number of the pathological parameters included in the cardio-metabolic risk (CMR) score.

The distribution of the variables and the number of the pathological parameters included in the CMR score, are reported in Table 3 and Fig. 1, respectively.

**Table 3:** Distribution of the cardiovascular, metabolic, and socio-familial parameters considered for the tool screening summary score.

Parameters	Number of patients (%)
Waist circumference/height ratio >0.5	255 (80.95%)
Body mass index (z-score) ≥2	276 (87.07%)
Pubertal stage (Tanner) >1	210 (66.25%)
Birth weight appropriateness (SGA or LGA)	47 (15.36%)
Gestational age (<37 week)	16 (5.76%)
Presence of maternal diseases during pregnancy	158 (50.97%)
Exposure to environmental factors during pregnancy in mother	74 (27.51%)
Exposure to environmental factors during pregnancy in father	11 (4.28%)
Presence of familiar/genetic risks	212 (68.61%)
Adiposity rebound (<6 years)	26 (11.76%)
Low socioeconomic familiar status	58 (24.17%)
Low aderence to MD Kidmed	111 (35.02%)
Gestational weight gain (≥10 kg)	197 (77.87%)
Cesarean section	115 (36.98%)
Non-exclusive breastfeeding	99 (32.78%)
Timing of cow’s milk introduction (<12 months)	40 (14.04%)
Timing of complementary feeding (<6 months)	56 (19.58%)
Infants’ antibiotics exposure	34 (12.78%)
Inappropriate bottle use	29 (14.01%)
Sleep duration below age-specific WHO recommendations	49 (16.44%)
Inappropriate physical level or sedentary	254 (83.28%)
Pathological blood pressure	29 (12.29%)

*MD* mediterranean diet, *SGA* small for gestational age, *LGA* large for gestational age.

As expected, a statistically significant difference was found between the two groups of patients with a high or low CMR score for the following parameters: WC (*p* = 0.007), BMI (*p* < 0.001), SBP (*p* = 0.004) and DBP (*p* < 0.001), and all metabolic parameters except for total and LDL cholesterol (*p* > 0.05), Table 4.

**Table 4:** Clinical parameters, biochemical and hormonal data of the enrolled patients.

	High CMR (score ≥7)	Low CMR (score < 7)	Total	*p*-value High Vs Low CMR
Waist circumference (cm)	87.20 ± 21.22	82.80 ± 18.50	85.52 ± 13.89	0.007
Weight (kg)	64.84 ± 20.69	64.59 ± 71.65	64.75 ± 47.31	0.965
Height (cm)	150.36 ± 14.00	148.26 ± 16.00	149.57 ± 14.80	<0.001
Waist circumference/height ratio	0.86 ± 0.35	0.72 ± 0.45	0.81 ± 0.39	0.212
Body mass index (kg/m2)	28.11 ± 5.31	25.58 ± 5.33	27.13 ± 6.54	<0.001
Body mass index (z-score)	2.73 ± 0.88	2.28 ± 1.3	2.56 ± 1.07	<0.001
Pubertal stage				
Tanner 1	68 (34.5%)	56 (46.7%)	124 (39.12%)	—
Tanner 2	66 (33.5%)	46 (38.3%)	112 (35.33%)	
Tanner 3	63 (32.0%)	18 (15.0%)	81 (25.55%)	
Gestational age	38.93 ± 7.93	39.13 ± 17.27	39.00 ± 1.90	0.404
Systolic Blood Pressure (mmHg)	113.72 ± 50.41	108.9 ± 52.38	112.03 ± 12.14	0.004
Diastolic Blood Pressure (mmHg)	70.91 ± 31.62	66.83 ± 30.99	69.41 ± 8.59	<0.001
Glycemia (mg/dl)	89.08 ± 33.66	86.17 ± 33.84	87.91 ± 9.54	0.015
Insulin (mcU/ml)	22.13 ± 19.26	13.26 ± 10.63	18.62 ± 16.88	<0.001
HOMA	4.97 ± 4.58	2.76 ± 1.91	4.11 ± 3.95	<0.001
TyG-index	8.29 ± 3.85	8.08 ± 3.97	8.21 ± 0.53	0.005
HbA1C (%)	5.26 ± 2.64	5.35 ± 2.71	5.29 ± 0.40	0.151
Total cholesterol (mg/dl)	155.29 ± 69.86	161.83 ± 78.80	157.77 ± 29.27	0.097
LDL cholesterol (mg/dl)	86.45 ± 45.08	81.03 ± 48.49	84.41 ± 32.62	0.222
HDL cholesterol (mg/dl)	50.38 ± 27.67	63.83 ± 36.84	55.54 ± 23.49	<0.001
Tryglicerides (mg/dl)	105.75 ± 68.80	81.92 ± 49.99	96.53 ± 53.55	<0.001

**Fig. 2:**
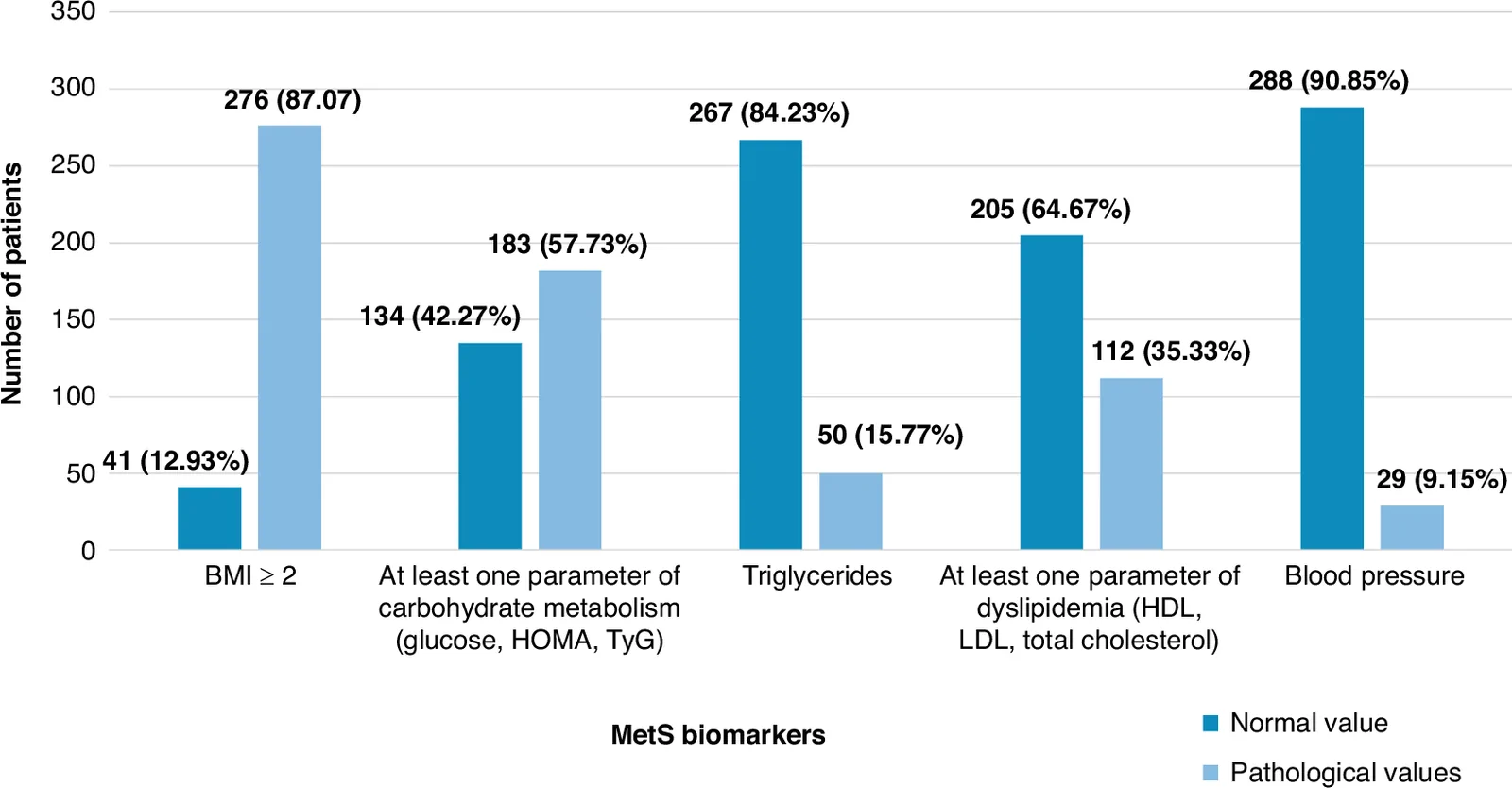
Number of the pathological parameters as regards metabolic syndrome (MetS).

The prevalence of MetS was 39.4%. In Fig. 2, the number of MetS parameters was reported.

The MetS severity z-score was correlated with the prevalence of MetS (Pearson’s *r* = 0.581). Linear association between the CMR tool and MetS severity z-score indicate a fair positive correlation (Pearson’s *r* = 0.334), suggesting that while the tool is useful for distinguishing the presence or absence of the condition, it is less sensitive to gradual variations in metabolic risk.

When considering a screening tool score over/above the median value of 7, along with patients presenting at least 3 out of 4 altered parameters of the MetS, the results demonstrated good sensitivity (76.8%), NPV (77.5%), accuracy (61.8%). However, specificity (52.1%), PPV (51.1%), +LR (1.603) and -LR (0.445) presented more limited performance. Table 5 presents all key diagnostic metrics of CMR tool along with their corresponding confidence intervals (CI). The diagnostic performance of the individual parameters included in the CMR assessment is reported in the Supplementary Material (Table S1).

**Table 5:** Key diagnostic metrics of cardio-metabolic risk (CMR) tool, with corresponding confidence intervals (CI).

Metric	Value	CI 95%
Sensitivity	0.768	(0.627, 0.843)
Specificity	0.521	(0.450, 0.591)
Accuracy	0.618	(0.564, 0.672)
PPV	0.511	(0.439, 0.582)
NPV	0.775	(0.702, 0.848)
+LR	1.603	(1.337, 1.924)
−LR	0.445	(0.314, 0.630)

95% CI were determined for all metrics computed from the contingency table using the Wald normal approximation, except for the likelihood ratios for which the log transformation was used.

The AUROC of screening tool score upon detection of cumulative biological dysregulation (pathological if at least 3 over 4 altered parameters) was 0.645 (95% CI 0.592–0.698).

## Discussion

We proposed a score to detect CMR in children and adolescents, based on their medical history, clinical characteristics, and lifestyle behaviors. A score ≥7 identified at-risk individuals and demonstrated good sensitivity in detecting MetS in the pediatric population. Despite its relatively limited sensitivity in this population, the tool nonetheless appears to hold considerable potential. It represents a non-invasive approach for the early identification of children at risk of cardiometabolic dysfunction, and may support both preventive strategies and personalized diagnostic and therapeutic interventions.

MetS is widely recognized as a significant risk factor for the development of both CVD and T2DM.^43–45^ Characterized by a cluster of interrelated conditions, such as central obesity, dyslipidemia, hypertension, and insulin resistance, MetS reflects a state of heightened metabolic dysfunction. Their constellation of abnormalities not only increases the likelihood of atherosclerosis and endothelial dysfunction, which are key contributors to cardiovascular events, but also accelerates the progression from IR to overt diabetes.^9^

During childhood and adolescence, increased fat mass together with hormonal and metabolic changes of puberty, such as heightened insulin resistance, contribute to the risk of developing MetS. As highlighted by de Lamas et al.^22^ pubertal progression is strongly associated with the consolidation of MS, and most children with early-onset MetS remain affected during puberty, markedly raising their likelihood of adult obesity, diabetes, and CVD.

As highlighted by Gurka et al.^12^ standard diagnostic definitions for MetS have certain limitations, as they only classify individuals as at-risk when specific thresholds are exceeded. This binary approach overlooks the idea that MetS may represent a continuum of risk. In fact, individuals with values just below the threshold in all five components might face a higher risk than those who exceed criteria in three components but have normal levels in the others. To address this, Gurka et al.^12^ proposed continuous MetS severity scores, enabling the monitoring of trends over time and the detection of variations based on clinical status. Several studies have demonstrated that these continuous scores correlate with unhealthy behaviors and elevated cardiovascular and metabolic risk.^46–48^ Moreover, they are associated with early markers of disease, including abnormal insulin and glucose levels, in both children and adults.^28,29^

In our pediatric population, pathological levels of the MetS severity z-score were observed in 62.15% of subjects. The MetS was correlated with the prevalence of MetS (*r* = 0.581), which was confirmed by the presence of at least three risk factors exceeding the age- and sex-specific thresholds, detected in 39.4% of participants.

These prevalence data are consistent with the recent systematic review and meta-analysis by Porchia et al.^49^ which analyzed 57 studies including a total of 27.923 participants. The overall prevalence of MetS among individuals with obesity varied widely across studies, ranging from 2.1% to 74.4%, with an average prevalence of 29.4%.

These alarming data underscore the urgent need for increased awareness and action regarding this issue.

Given the significant resources and time involved in diagnosing MetS, conducting large-scale screenings is often unfeasible. As a result, there has been a shift toward more practical and affordable methods, such as self-reported questionnaires. These tools are advantageous because they are straightforward to use, require minimal time, eliminate the need for blood samples or trained personnel, and incur lower costs.

Among adults, many self-administered questionnaires have demonstrated strong predictive performance. For instance, the American Diabetes Association created a tool^50^ to assess the likelihood of developing T2D, showing reasonable agreement with clinical diagnoses (sensitivity 79%; specificity 65%). In Greece, the Finnish diabetes risk score (FINDRISC)^51^ was found to effectively detect MetS with high sensitivity (89%–98%), though specificity remained low (14%–37%).

Considering that the development of chronic cardiometabolic diseases results from the combination of risk factors across prenatal, childhood, and adulthood stages, we proposed a tool for the pediatric population that takes into account both modifiable and non-modifiable risk factors for the development of MetS, CVD, and T2D.

Our results show that, although the proposed score achieves good sensitivity (76.8%) and a satisfactory PNV (77.5%), specificity (52.1%) and positive predictive value (51.1%) remain moderate, leading to a non-negligible number of false positives. While this represents a limitation for its use as a diagnostic or confirmatory test, as it is less sensitive to gradual variations in metabolic risk, it does not diminish the potential value of the tool as a simple, non-invasive first-level screening aid, designed to maximize the identification of at-risk individuals without requiring blood tests, while minimizing missed cases. The moderate discriminatory ability (AUROC 0.645) further underscores its value, not as a definitive diagnostic tool but rather as a first-level risk assessment for stratifying the pediatric population and identifying individuals who may benefit from further evaluation through blood testing, thereby reducing unnecessary consultations and costs while facilitating earlier diagnosis. However, in cases where MetS is already suspected, this tool is not intended to replace medical consultations or follow-up assessments, but rather to help identify those at potential risk.

The authors acknowledge the limitations of this study. First, the number of enrolled participants is relatively small, and further validation studies will be needed to confirm and enhance the tool’s accuracy. Secondly, the questionnaire did not consider certain factors that may be relevant to increased risk, such as ethnicity, and could therefore be improved by incorporating additional data.

Secondly, the participants were recruited from a clinical setting, where the prevalence of MetS is higher than in the general population. This may have influenced the predictive values of the tool, and although sensitivity and specificity are intrinsic test characteristics, some evidence suggests that prevalence may also affect these measures. Therefore, our findings should currently be interpreted with caution, as they may not be directly generalizable to the general population and will require validation in larger, population-based cohorts.

Additionally, physical activity and sleep duration were obtained from parent- or caregiver-reported information during clinical anamnesis. Although self- or proxy-reported measures may be subject to recall bias, previous studies have shown that parent-reported lifestyle information in children provides acceptable reproducibility and validity when compared with objective measures, especially in large-scale or clinical settings where accelerometry or actigraphy are not feasible.^52,53^ Moreover, categorization of these variables according to WHO age-specific guidelines increases their interpretability and comparability across studies.

Finally, our tool was developed and evaluated using a single, pre-specified pediatric MetS definition based on fixed, clinically interpretable thresholds, demonstrating its reliability through key diagnostic metrics: accuracy, sensitivity, specificity, and AUROC, with appropriate confidence intervals. These measures directly reflect the discriminative ability and robustness of the score in the defined context. We did not perform cross-validation against other widely used pediatric definitions^33,35,37^ or continuous scores.^27^ Consequently, performance estimates and case classifications may not be directly comparable with percentile-based or cohort-referenced approaches, and some reclassification of individuals would be expected under alternative frameworks. While cross-definition validation could improve generalizability, we believe the current diagnostic analysis provides a solid foundation for initial use and represents a prudent intermediate step before possible future extensions, which we identify as a priority for future work.

## Conclusions

The proposed CMR score demonstrated good sensitivity and satisfactory overall accuracy, although its specificity was more limited. The accuracy data support its value as a non-invasive, first-level risk assessment tool for the early identification of cardiometabolic risk in children, rather than as a definitive diagnostic tool. By enabling the timely recognition of at-risk individuals, the CMR score can support prompt interventions and more personalized diagnostic and therapeutic strategies, ultimately saving time and reducing healthcare costs through earlier diagnosis.

## Supplementary information


Supplementary information


## Data Availability

The data that support the findings of this study are available within the article.
