# Supplementary material for: A non-invasive tool for the early identification of children at risk of cardiometabolic dysfunction: data from the PODiaCar project
Source: Pediatr Res. 2025 Dec 3;100(1):331–9. doi: 10.1038/s41390-025-04603-y (PMC13524810; doi:10.1038/s41390-025-04603-y)
Supplement: Supplementary file 1 [file 41390_2025_4603_MOESM1_ESM.pdf]

Table S1. Sensitivity, specificity, and accuracy of the individual parameters used to create the cardiometabolic risk (CMR) assessment were evaluated against the total risk score. For each parameter of the tool's total score, a contingency table was constructed with two dichotomous variables: whether the overall score was greater than or equal to (or less than) the median value of 7, and whether the parameter was classified as pathological (or normal). 'True positives' were defined as subjects with a total tool score ≥7 and a pathological value or condition for the parameter, as described in Table 1. Sensitivity, specificity, and accuracy were then calculated from these tables.

Parameters	Sensitivity	Specificity	Accuracy
1. Gestational age	0.688	0.412	0.426
2. Presence of maternal cardiometabolic diseases during pregnancy	0.772	0.585	0.678
3. Exposure to environmental factors during pregnancy in mother	0.865	0.490	0.577
4. Exposure to environmental factors during pregnancy in father	0.545	0.405	0.410
5. Birth weight	0.766	0.437	0.486
6. Mode of delivery	0.783	0.515	0.612
7. Gestational weight gain	0.701	0.582	0.656
8. Familiar/genetic risks	0.693	0.610	0.666
9. WC/Ht ratio	0.639	0.597	0.631
10. Adiposity rebound	0.923	0.436	0.476
11. BMI (z-score)	0.652	0.805	0.672
12. Pubertal stage	0.668	0.524	0.612
13. Socioeconomic familiar status	0.845	0.463	0.533
14. Adherence to MD_Kidmed	0.847	0.544	0.650
15. Exclusive breastfeeding	0.778	0.491	0.580
16. Timing of cow's milk introduction	0.575	0.404	0.426
17. Timing of complementary feeding	0.857	0.464	0.533
18. Infants' antibiotics exposure	0.912	0.445	0.495
19. Inappropriate bottle use	0.897	0.438	0.479
20. Duration of sleep	0.735	0.433	0.479
21. Physical level or sedentariness	0.677	0.746	0.691
22. Blood pressure	0.793	0.427	0.461
